# Identifying multilevel and multisectoral strategies to develop a Theory of Change for improving child and adolescent mental health services in a case-study district in South Africa

**DOI:** 10.1186/s13034-022-00484-9

**Published:** 2022-06-18

**Authors:** Gbotemi B. Babatunde, André Janse van Rensburg, Arvin Bhana, Inge Petersen

**Affiliations:** Centre for Rural Health, School of Nursing & Public Health, University of KwaZulu-Natal, Durban, South Africa

**Keywords:** Theory of Change, Child and adolescent mental health, Mental healthcare plan, Collaborative planning

## Abstract

**Background:**

The lack of child and adolescent mental health (CAMH) policies and implementation plans constitute major barriers to CAMH services in low resource settings. Engaging with on-the-ground stakeholders to identify possible contextually appropriate strategies for developing a CAMH collaborative system and inform CAMH plans and policies is important to ensure that resultant policies and plans are feasible and appropriate. Together with key stakeholders across multiple sectors, this study aims to (i) co-identify causal factors and potential strategies to overcome bottlenecks in one district in SA as a case study; and (ii) Co-develop a Theory of Change (ToC) for increasing access to CAMH services within the resource constraints of a remote resource-scarce district as a case study.

**Methods:**

A participatory workshop was held with key stakeholders (n = 40) from the Departments of Health (DoH), Basic Education (DBE), and Social Development (DSD) and three community-based organisations offering CAMH services in the district. The stakeholders identified context-specific causal factors and possible strategies to address the bottlenecks in the workshop. All the factors identified in the workshop were compared and consolidated. A ToC map was developed based on the data obtained from the workshop. The ToC was further refined by conducting a follow-up virtual workshop with stakeholders (n = 15).

**Results:**

Mapping out the strategies identified in the workshop facilitated the development of a ToC model for the resource-scarce context. Key multilevel and multisectoral task-sharing strategies emerged in support of the development of a collaborative system of care that includes the development of (i) community awareness programs and user-friendly CAMH psychoeducation and screening tools to strengthen mental health literacy and facilitate early identification at the community level; (ii) an intersectoral working group to facilitate intersectoral collaboration (iii) a functional district CAMH referral system, (iv) youth-friendly CAMH care packages.

**Conclusions:**

In scarce-resource contexts, it is feasible to work collaboratively with key stakeholders across multiple sectors to identify feasible multilevel and multisectoral strategies that can be used to develop a ToC for improved access to CAMH services within a task-sharing approach.

## Background

Child and adolescent mental health (CAMH) services and interventions remain underdeveloped in many low- and middle-income countries (LMICs). The available CAMH services in many of these countries are marked by the lack of polices, low political priority, low financial investment, shortage of human resources and infrastructure, lack of adequate coordination and intersectoral collaboration [24, 26, 29, 33]. To address these issues, it is important to integrate CAMH services into primary health care and different community platforms such as schools and homes [6, 27]. Also, consistent engagement between stakeholders from the different sectors providing CAMH services is equally imperative to ensure a well-coordinated and effective system of CAMH care [4].

According to Guise et al. (2013), researchers must engage with stakeholders within key sectors and other community organisations in participatory planning to identify contextually appropriate strategies that could be used to develop a collaborative system of care. Haldane et al. [20] also stated that the optimisation of health interventions and effective collaboration or partnership between key sectors (health, education, and social development), community-based organisations, and the community at large could be facilitated through a community participatory approach. This approach has proven effective in addressing challenges encountered when planning and implementing health interventions. These challenges include power relations, trust-building, and understanding the socio-economic, and political contexts of the proposed initiatives [20]. This is also necessary to ensure adequate mobilisation and utilisation of the available resources and obtain stakeholders' buy-in [20, 25]. Hence, a participatory approach is fundamental to developing a collaborative system of care to improve CAMH services due to the range and characteristics of the key role players, service providers, and service users.

### Theory of Change

Theory of Change (ToC) is a highly recommended theory-based participatory approach that has recently been used in designing and evaluating complex mental health interventions globally [12, 14]. It provides detailed descriptions of how specific interventions contribute to the desired outcomes by presenting the causal pathways, the link between inputs, outputs, and activities and how they produce the expected impact [18, 40]. It helps reveal fundamental factors that predict the success and failure of the interventions [11], and the participatory process assists stakeholders to articulate their ideas and identify their different priorities. Also, De Silva et al. (2014) highlighted that the ToC process provides ways of identifying and addressing challenges that emerge while designing interventions.

If a ToC is effectively developed, it can serve as a framework for service planning and implementation [42]. According to Hernandez & Hodges [21], ToC provides a context for incorporating a community's shared beliefs that informs the expected outcomes and the strategies suitable for achieving the desired outcomes. Therefore, a ToC map is usually developed by consulting with a wide range of stakeholders across different sectors through workshops and meetings to ensure all underlying community factors and the different sectors' goals, routines, strategies, budget, and resources are adequately incorporated [13]. Moreover, the inability to achieve consistent impacts from community interventions often stems from the failure to establish the connection between the target population, their shared beliefs, desired outcomes, and strategies to achieve them [43]. ToC has been used in designing mental health interventions in some low and middle-income countries (LMICs), including Ethiopia, Nepal and South Africa [1, 12, 14, 37]. In this study, we used ToC as a framework underpinning the development and evaluation of a district child and adolescent mental health care plan.

Using one district in the province of KwaZulu-Natal as a case study for the development of an integrated multisectoral system of CAMH care, formative work initially involved a situational analysis of existing CAMH resources and services and key stakeholder interviews, reported elsewhere [5, 7]. This study revealed six major bottlenecks (presented to CAMH services that cut across all the different sectors in the district. They included: poor governance structures (lack of intersectoral collaboration/liaison forum); poor identification, screening, and assessment procedures; poor referral pathways; specialist vertical services; limited community-based CAMH interventions, and limited CAMH promotion and awareness. Building on these findings, the present study aimed to i) Co-identify causal factors and potential strategies with key stakeholders that could be feasibly implemented to effectively address bottlenecks identified during the formative phase; and ii) Co-develop a Theory of Change (ToC) to feasibly increase access to CAMH services based on the identified strategies that could be used for the development of a district mental health care plan.

## Methods

### Study context/site

The study site was the Amajuba District Municipality, a district in the northwest part of the KwaZulu-Natal province and serves as a gateway into the Mpumalanga and Free Stae provinces. It is a small district covering about 7102 km^2^ which is only 8% of the province's geographical area. The district has a population estimate of about 556,580, and about 51% of the population are children and adolescents (Amajuba district profile, 2020). The district comprises mainly rural and peri-urban communities, including Charlestown, Newcastle, Madadeni, Osizweni, Dannhauser, Hattingspruit, Utrecht and Kingsley, within three sub-districts: Newcastle, eMadlangeni and Dannhauser (Amajuba district profile, 2020; Amajuba District Municipality, 2017; Govender et al. [45]. The district has a total number of 127,000 households, 12.3% of these households live in informal settlements (shacks), and about 788 are child-headed. About 416,000 people live in poverty due to high unemployment rates despite the documented increase in people with 'matric' or higher education in the district. 

Amajuba district is resource-constrained, with few health professionals, including mental health specialists, to provide adequate health care services for the populace Govender et al. [45]. The Amajuba District was selected as a case study site, given that the study builds on existing relationships and services that have been developed through the Mental Health INTegration (MhINT) program with the KwaZulu-Natal Department of Health [38]. The MhINT program is a system strengthening intervention that provides technical support, training, and guidelines to enable the integration of mental health into primary health care for adults with common mental disorders using a task-sharing approach [38].

This study also leveraged the district's existing mental health and disability forum. The forum was headed by the district mental health coordinator and included service providers within the Department of Health, community-based organisations (CBOs) representatives, social workers from the Department of Social Development, directors from the Department of Education and some community/religious leaders. This forum met monthly to discuss mental health and disabilities issues within the community. The committee members are prominent leaders within the departments and community champions who can facilitate collaboration, allocation of resources, implement interventions and influence change within the different departments and across the different levels of care. The committee had previously requested CAMH interventions during their engagements with the MhINT team and also displayed their readiness to promote change through their active participation during the workshops. 

Our situational analysis Babtunde et al., [46], which involved engagements with different stakeholders, including service providers and caregivers, revealed the commonly reported CAMH conditions in the district. They included intellectual disabilities, attention deficit hyperactivity disorder (ADHD), adjustment disorders, depressive disorders, and substance use. The situational analysis also revealed a severe shortage of specialised CAMH services in the district, poor integration of CAMH services into primary health care and the absence of clear referral pathways. During our initial engagements with some of the forum members, they mentioned that they visited schools routinely and were often consulted to address mental health-related crises such as substance abuse and suicide within schools. Despite limited resources and expertise, community based organizations (CBOs) were found to be active players in CAMH promotion and awareness in the district.

### Research approach

This study employed a participatory action research (PAR) approach [8, 30]. According to Baum et al. (2006), PAR is a collective self-reflective process undertaken by researchers and participants to understand and improve health practices through specific actions to impart social change.

The study adopted a three-step process (presented in Fig. 2) which included (1) conducting an initial participatory workshop to co-identify causal factors and potential strategies to address the identified bottlenecks with key stakeholders, (2) developing a ToC model, (3) presenting the ToC model back to the stakeholders and obtaining their feedback in a follow-up workshop.

### Participants

Research participants were purposively identified according to their positions in the departments of health, social development and education. Stakeholders interviewed at the earlier phase of the study were invited to attend the workshop. The first participatory workshop involved forty (40) stakeholders from the departments of Health, Basic Education, Social Development, and three CBOs providing CAMH services in the district. The participants included representatives from community-based organisations, managers including the district mental health coordinator, school directors, and service providers, including nurses, clinical psychologists, occupational therapists, pharmacists, social workers, and educators (high school and primary school teachers).

## Step 1: Participatory workshop to identify causal factors and potential strategies

Prior to the first workshop, one of the researchers (GBB), made several visits (between February and September 2019) to the district to meet with key stakeholders in all the sectors providing CAMH services. Invitation letters were sent out to all stakeholders that were interviewed during the formative phase of the study. Also, follow-up visits were made prior to the workshop to remind the participants of the workshop. 

We followed the workshop procedures described by Breuer et al. (2014) to ensure a reasonable degree of participation and rigor. Researchers engaged the participants' problem-solving capabilities to facilitate active involvement within the workshop, drawing on their vast knowledge and experiences of providing CAMH services in the district. The workshop was specifically organised to achieve predetermined outcomes [36], in this case, to co-identify collaborative strategies to improve CAMH services in the district.

The workshop was conducted in a private space at a venue centrally accessible to all the participants. All participants indicated they were proficient and comfortable with English language; for this reason, all discussions were held in English. The workshop facilitator, experienced with conducting participatory workshops, opened the meeting and formally introduced all the participants. The research team introduced the workshop agenda and facilitated an agreement on the length of the workshop.

Prior to the discussions, GBB, presented the findings of the formative studies to acquaint the participants (key stakeholders across multiple sectors) with an up-to-date status of CAMH services in the district as well as the identified bottlenecks. This was necessary as the workshop's goal was to co-identify causal factors and potential strategies to address the bottlenecks identified, with the view of developing a ToC model that could inform a district CAMH plan. The participants provided feedback and validated the findings of the formative study.

Participants were divided into three structured multidisciplinary groups to ensure a mix of stakeholders from the various sectors. The inclusion of different categories of stakeholders in each group to engage with the bottlenecks eliminated the tendency of power imbalance as every member was considered equal, and their contributions were valuable to identifying viable strategies in the workshop. Workshop materials (flip charts, markers, pens, and coloured papers) were given to the participants to make notes. Each group was asked to outline the causes of the bottlenecks and identify potential strategies to address them. Two bottlenecks were randomly allocated to each group, and the group discussions lasted for an hour.

Following the small group discussions, each group provided feedback to the larger group during the plenary session—which steered further large group discussions around the similarities and differences in the group discussions. The facilitator moderated the sessions and kept a tab on time. The stakeholders' participatory workshop lasted for 4 hours. Member checking was done at the end of the plenary session, where the workshop facilitator summarised the key points from the discussions and asked all the participants to confirm these. Three researchers (IP, AV, and GBB) were observers of the workshop but were not active participants in the discussions. The notes taken by participants in each group were consolidated and analysed after the workshop. The audio recordings were transcribed verbatim and compared with the notes taken by the participants and researchers. This corroborated the findings of the study.

## Step 2: Development of the Theory of Change (ToC) map

In the second stage, GBB and AV drafted the ToC map for CAMH services in the district by combining the data obtained from the workshop and input from the formative study detailed elsewhere [5, 7]. The researchers used this information to identify medium- and long-term outcomes, interventions/strategies, and causal pathways that could lead to the intended outcome as well as indicators to measure the degree of change achieved—thereby creating a different approach to developing a ToC map. The two researchers (GBB, AV) met weekly to brainstorm the medium-term outcomes, the necessary strategies to achieve them, and the connecting pathways. The researchers reviewed and categorised all the field materials provided by the participants in the first workshop and also referred to the findings of the formative studies to identify all the elements of the map. GBB and AV consulted with IP to refine the initial ToC, and this provided the researchers with an opportunity to cross-examine the outcomes and strategies as well as identify key indicators to measure the change in each of the medium outcomes before agreeing on the final ToC map.

## Step 3: ToC improvement and refinement

The third stage of the ToC development process involved obtaining feedback on the ToC map and narrative from the research team and a follow-up workshop with the stakeholders. All the 40 stakeholders present at the first workshop were invited through emails and phone calls. An online follow-up workshop was conducted in August 2021 due to COVID 19 restrictions to present the consolidated findings and ToC map. Fifteen stakeholders' representatives from the Health, Social Development and Education departments who were part of the first workshop attended. The remaining 25 participants could not attend due to their unavailability and limited access to the required infrastructure to attend a virtual workshop. During this workshop, GBB presented the ToC map and allowed the stakeholders to provide feedback. The stakeholders were encouraged to validate, refine or realign the draft ToC map to what is attainable in their context.

### Data collection

The data for this study was obtained mainly from the workshops materials (audio recordings, participants and researcher notes). The plenary session of the first ToC workshop was audio-recorded, and notes were taken by the first author. These notes were checked against the audio file for completeness. In the second (follow-up) workshop, the first author took notes, and the virtual meeting conducted via Zoom was recorded. The first workshop lasted 5 hours, and the follow-up virtual workshop lasted for 1 hour 30 mins. Data from the ToC workshop, several drafts of the ToC maps, review from the research team and feedback from the stakeholders lead to the final ToC map.

### Data analysis

The data was analysed following the structured, systematic steps of thematic analysis [10]. The first step in the analysis process involved familiarising oneself with the data set, which was done by reading through the field notes and the transcripts several times. The second step involved comparing and consolidating the field notes taken by the participants in each group and coding the relevant features of the data. The third step involved identifying themes and sub-themes. The fourth step involved reviewing and categorising all the themes. Finally, the themes were redefined, and the generated sub-themes were clustered into meaningful categories. To ensure reliability, two of the researchers (G.B.B. and AV) independently coded all the data and met regularly to discuss data interpretations and key findings to ensure consensus and saturation. All differences in coding were resolved through these consensus discussions. A third researcher (IP) was consulted if GBB and AV could not reach a consensus.

### Ethics

Permission to conduct the study was received from the provincial Departments of Health and Basic Education. Also, ethical approval for the study was obtained from the Biomedical Research Ethics Committee, Faculty of Health Sciences, University of KwaZulu-Natal. Reference number BE098/18. All participants were given information sheets, and all provided written informed consent before participating in the study.

## Results

**Fig. 1:**
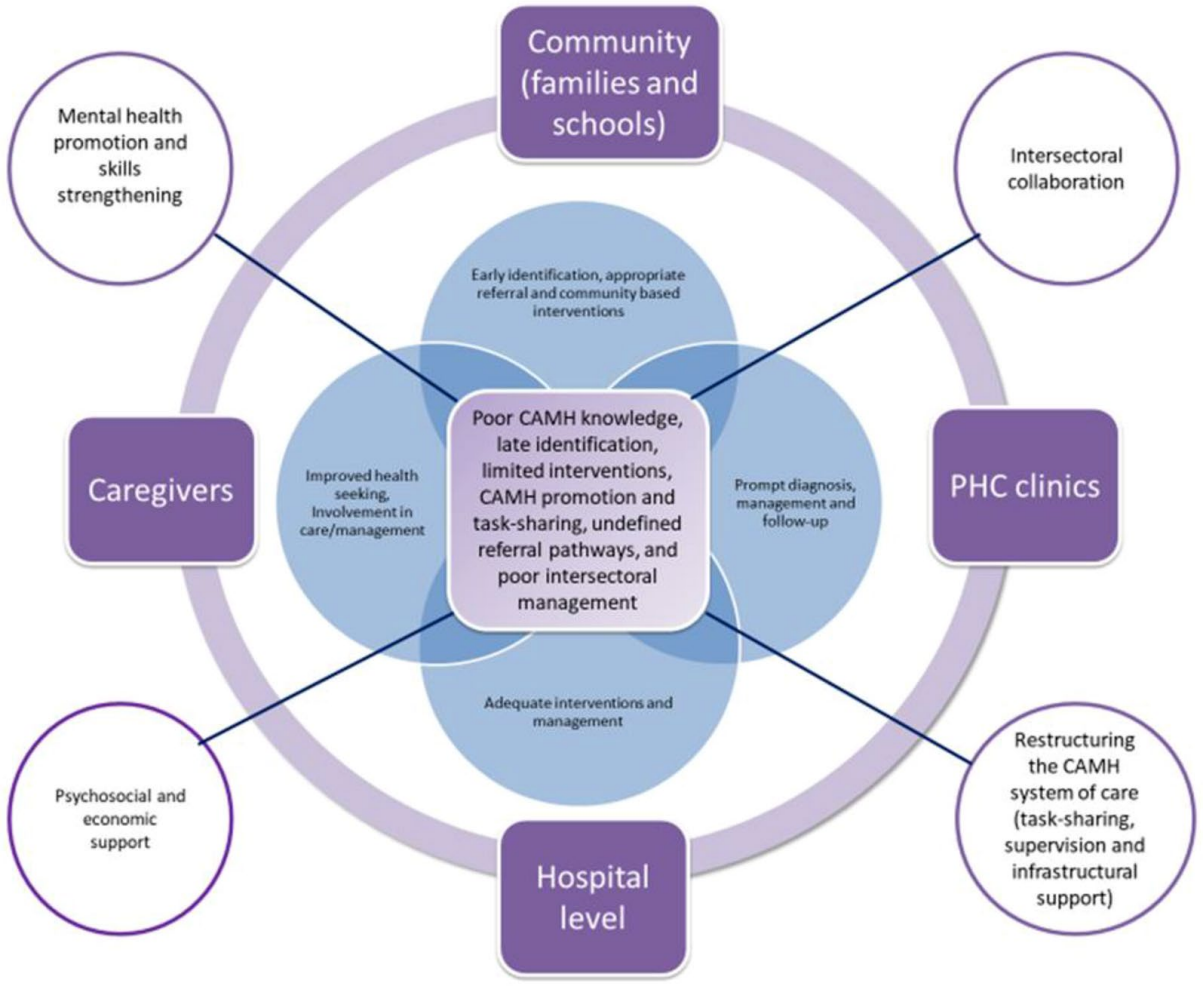
Bottlenecks and multilevel interventions

As stated earlier, participants generated causal pathways focusing on system bottlenecks and potential strategies during the ToC workshop. Hence, the findings have been categorised into six sub-themes (the identified bottlenecks) that report on "Causal factors" and "Possible strategies" presented in Table 1 below. Figure 1 illustrates the link between the bottlenecks, possible strategies, and the different levels of CAMH care (community, primary health care (PHC), caregivers, and hospital). It also presents solutions specific to each level of care (within the set) and strategies that are cross-cutting (pointing out of the set).

**Table 1:** Summary of workshop data

Group (s)	Why bottlenecks—Causal factors.	What can be done—possible solutions.
1	Poor identification/screening and assessment Lack of training for stakeholders Lack of community education Family system—Mothers not trained to pick up the red flags despite having the road to health cards, grandmothers raising children, parent denial Limited CAMH facilities and resources (human resources and Screening tools)	Educate caregivers and encourage active involvement Conduct routine awareness campaigns through community structures, e.g., NPOs and Ward Based Primary Health Care Outreach Teams (**WBPHCOTS**) Develop user-friendly screening tools, train educators to identify and do basic screening in schools and provide basic community support
	Inappropriate referral pathway Poor referral structures Limited training specifically within the educational sector and NPOs Lack of knowledge on structures that exist in some facilities, e.g., conferencing (communication structures) for DOE/DOH Poor communication between stakeholders	Adequately define referral pathways Communicate all relevant information to stakeholders Implement referral policies for each department
2	Limited CAMH promotion and awareness CAMH not prioritized Lack of funding/budget allocation Lack of training and campaign materials Lack of community dialogues around CAMH and awareness programs Lack of structured and consistent awareness campaigns	Consistent awareness campaigns Prioritizing awareness/pro-active measures rather than an immediate reaction to an unfortunate event Prioritize staff training Engage in small/less costly awareness campaigns more often Use of school health nurses, NPOs and counselors in schools to create awareness among learners and destigmatize mental health all the time
	Community-based interventions A poor intersectoral working relationship Inadequate attention to CAMH interventions Lack of dialogues with young people	An improved working relationship between the departments Availability of recreation facilities Involve youth in decision making Make the community aware of chill rooms in clinics Establish more buddy with youths (mentorship programs)
3	Poor management structures The low priority given to CAMH by the government Management attitude to implementing procedures at all levels (lack of understanding within each sector and intersectorally) Lack of budget allocation Poor planning	Intersectoral CAMH liaison forum Advocacy for budget allocation in all the departments The different departments need to inform and educate each other on the different policies Collaborative activities such as designing and implementing CAMH interventions
	Task sharing Limited specialists Increasing workload Shifting of responsibilities (within the different departments) Shortage of school health team	Increase CAMH specialists Mentorship and specialist supervision Train staff in all departments (educators, nurses, school health team) Managers must ensure that they take up the responsibilities to deliver adequate CAMH services

### Causal Factors and possible strategies for poor identification, screening and assessment procedures

Four main causal factors of poor identification, screening and assessment procedure emerged during the analysis—(i) Lack of training on CAMH for health workers and educators (ii) Lack of community mental health education programs, particularly CAMH, (iii) family factors, including mothers' lack of knowledge on CAMH and their inability to interpret red flags on their children's road to health cards, (iv) children are cared for by grandmothers with low literacy level and limited resources and (v) parental denial—many parents struggle to accept their children's conditions, (vi) Limited CAMH facilities and resources, particularly the shortage of human resources in the district and difficulties associated with the availability of screening tools, lack of training on their use, and the length and time required to administer the tools. The possible strategies highlighted by the participants included (i) educating parents on how to identify growth/ developmental deviations in their children before attaining school age and facilitate the active involvement of parents in the management of children with CAMH challenges (ii) conducting routine awareness campaigns in the community—the participants mentioned that effective community awareness campaigns could be achieved by creating outreach teams and involving the relevant non-governmental/non-profit organisations (NPOs) in the district (iii) the need to develop user-friendly screening tools and the need to train stakeholders particularly educators and health workers at the primary health care (PHC) level to use the screening tools.

### Causal factors and possible strategies to address inappropriate referral pathways

Based on the preliminary studies, inappropriate referral pathways were identified as a major weakness of CAMH services in the district [5, 7].

**Fig. 2:**
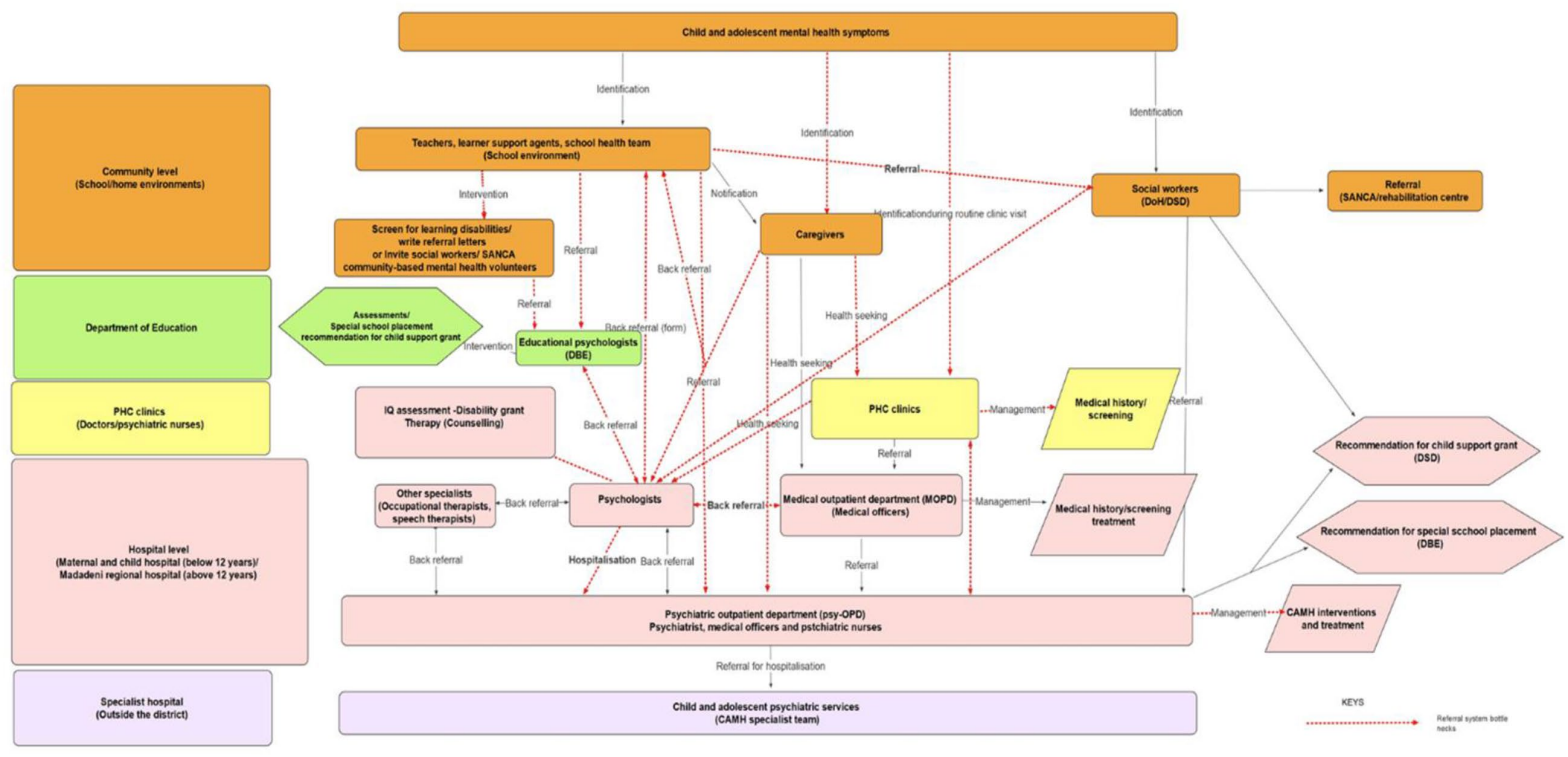
Existing referral pathways in the district

The referral pathways illustrated in Fig. 2 described in detail elsewhere [5, 7] revealed that the majority of the CAMH cases identified in the district were referred to the two clinical psychologists based at the hospital, who provided psychological interventions and referred to other specialists such as the occupational therapists, speech therapists and the psychiatrist for further interventions. Many of the referrals to the clinical psychologists were for learning disabilities that should have ideally been referred to educational psychologists. The clinical psychologists thus reported that many of the cases were referred back to the educational psychologists at the Department of Education. This illustration reveals the shortcomings of the CAMH referral system in the district and the need to define the referral pathways clearly**.**

Participants reported four main factors responsible for the inappropriate referrals of CAMH cases in the district. They include poor referral systems, limited training on CAMH identification and referral, lack of knowledge on the existing referral systems and poor communication between stakeholders within each sector and between sectors. According to the participants, the lack of adequately defined multilevel/multisectoral referral pathways and lack of training on CAMH identification and referral, particularly for educators, were major challenges impeding access to CAMH services in the district. Hence, they highlighted the need for capacitation in screening and identification of cases in need of referral and clearly defined CAMH referral pathways that incorporate all the relevant departments and sites of care. Also, the need to develop referral guidelines and training to disseminate the necessary information included in the referrals guidelines was highlighted.

### Causal factors and identified strategies to address limited CAMH promotion and awareness

Participants ascribed the general lack of knowledge on CAMH in the district to limited CAMH awareness programs in the community. Stakeholders from DOH reported that the lack of community dialogues around CAMH is reflective of the low priority given to mental health. Schools are visited mostly after adverse events (such as suicide) to provide therapy and educate learners in mental health literacy. Stakeholders from the NPOs mentioned that they conducted mental health campaigns in schools and communities, although inefficiently due to the lack of training and campaign materials. Also, stakeholders from DBE mentioned that campaign programs were organised on days allocated globally to the recognition of CAMH conditions. Participants suggested that CAMH awareness campaigns should be more frequent and not only according to the health calendar or immediate reaction to an unfortunate event. The need to train school health nurses, NPOs, and learner support agents in schools to create awareness among learners and organise cost-effective awareness campaigns to destigmatise mental health in the community was mentioned.

### Causal factors and identified strategies to address limited community-based CAMH interventions

Participants reported that the poor working relationship between the Departments of Health, Basic Education, and Social Development contributed in a major way to the limited community-based interventions in the district. This was despite that the three departments jointly created a disability forum where representatives from the departments met monthly to discuss issues about disability in the district. Also, there was a mental health disability forum within DOH where stakeholders meet to discuss the mental health challenges in the district. However, these forums are not solely dedicated to CAMH discussions. The participants mentioned that the NPOs were part of these forums as they served as key informants and communication channels between government and communities. However, inadequate attention was paid to developing multisectoral CAMH interventions and initiating dialogues with young people in the district. Suggestions raised to address these issues during the workshop included: an improved working relationship between the departments specifically to address CAMH issues, provision of recreation facilities for young people, and the need to create awareness on the availability of youth-friendly facilities such as "chill" rooms in the clinics. The need to create dialogical spaces for young people, design mentorship programs, and involve them in decision making while designing CAMH programs were also mentioned.

### Causal factors and identified strategies to address poor management structures

The preliminary studies revealed the lack of a multisectoral CAMH management structure and poor management structures for CAMH within each of the departments as major weaknesses of CAMH services in the district [5, 7]. Participants in the workshop mentioned that the causal factors include the lack of a functional CAMH management structure which results in poor planning, failure to implement adequate procedures for CAMH service delivery, and the inability to facilitate budget allocation. Therefore, the identified strategies include the need to build a functional intersectoral liaison forum to facilitate joint advocacy for budget allocation and collaborative activities for the delivery of CAMH services were suggested. The need to communicate the existing policies relevant to the delivery of CAMH services in each department was further emphasised.

### Causal factors and identified strategies to address limited specialists and vertical services

The shortage of specialists, including psychiatrists, psychiatric nurses, educational and clinical psychologists and the lack of dedicated CAMH specialists were highlighted as causal factors. The suggested strategies highlighted by the participants included specialist supervision and mentoring of non-specialists. According to the participants, these strategies are necessary to reduce the workload and facilitate prompt access to CAMH services in the district. The participants further stated that increasing the number of CAMH specialists and support staff across all the departments is imperative. According to the participants, it is important to equip staff at all levels (community, school, primary healthcare, and hospitals) with basic skills and knowledge required to deliver adequate CAMH services. Additionally, the participants mentioned that managers at the different departments must ensure that staff are adequately monitored to provide effective and prompt CAMH services in the district.

## Results from stage two

### Development of theory of change map for CAMH services

**Fig. 3:**
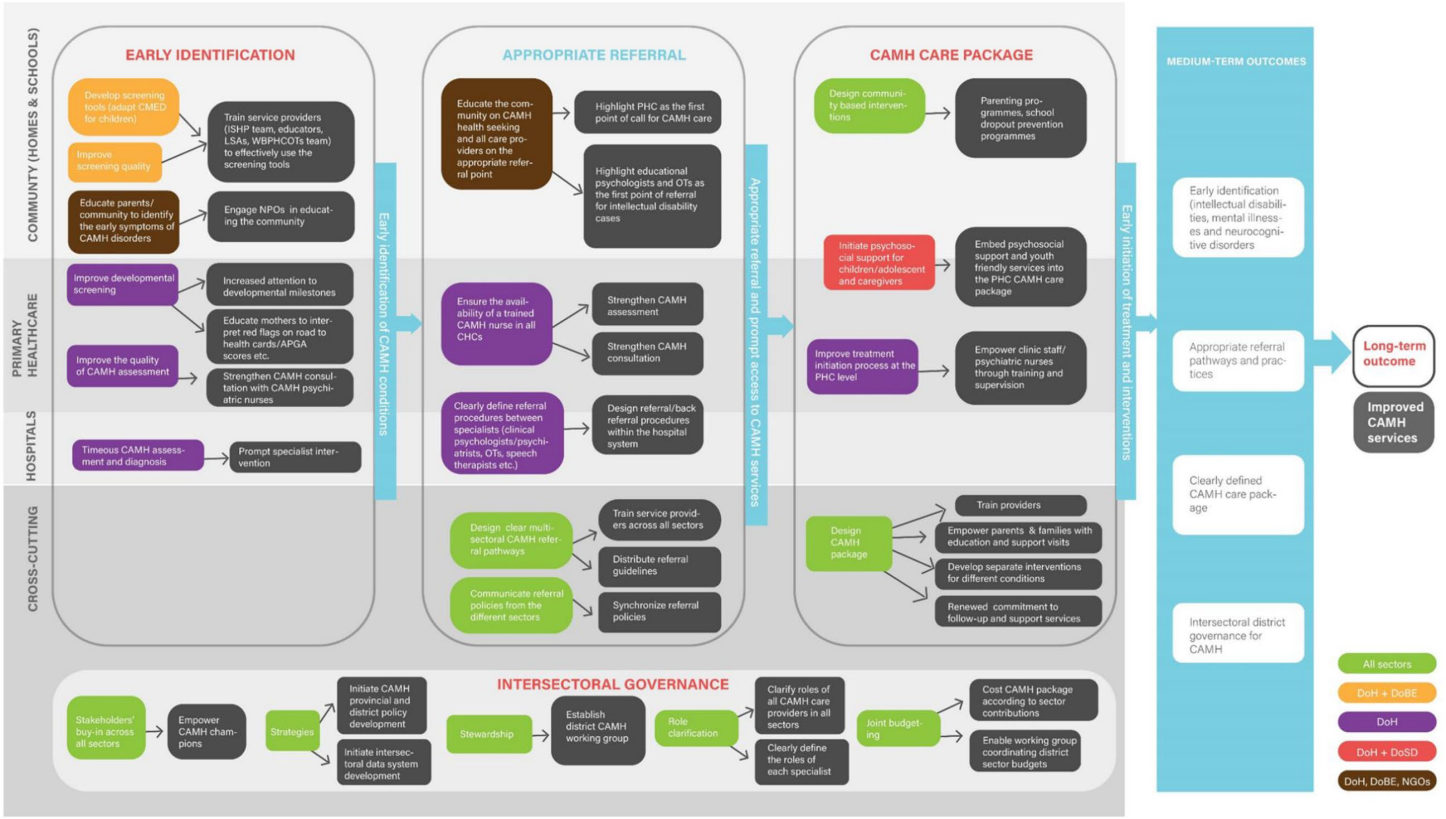
ToC map: collaborative strategies for CAMH services

The ToC map (Fig. 3) maps out the medium-term outcomes, the necessary activities (collaborative strategies for CAMH care) to achieve each medium-term outcome, and the causal links connecting medium-term outcomes to the long-term outcomes. The ToC map also presents multilevel and multisectoral strategies for each medium-term outcome.

Medium-term outcomes include early identification of CAMH conditions, appropriate referral and prompt access to CAMH services, development of CAMH care packages (for early initiation of treatment and interventions), and intersectoral governance. The long-term outcome was identified as improved CAMH services. The medium-term outcomes cut across different levels of care, including the community (school and home environment), primary health care, and hospitals. Furthermore, interventions and strategies for each level of care were mapped out, and some cross-cutting interventions were also identified.

A detailed description of the relationship between all the variables, inputs, activities and indicators are presented in Table 2 (ToC log frame). The ToC map and log frame present possible ways through which the CAMH system in the district can be strengthened.

**Table 2:** Theory of Change log frame

Objectives			Inputs	Outputs			Outcomes		
Description		Sub-objectives	Activities	Description	Indicators	Data sources	Description	Indicators	Data sources
1a.Early Identification	Strengthen the identification and screening of CAMH conditions	Develop CAMH tools (adapt CMED for children) and equip teachers and learner support agents (LSAs) with the skills and knowledge required to use the screening tool	(a) Train teachers and learner support agents on CAMH identification and use of screening tool (b) Distribute screening tools and guidelines	(a) Equipped teachers and LSAs (b) Functional tools and guidelines	(a) The number of schools covered (b) Number of teachers and LSAs trained (c) Number of tools and guidelines distributed	(a) completed training registers (b) signed sheets for tools and guidelines received	Teachers and LSAs are equipped to identify CAMH conditions and screen within the school environment	Improved CAMH screening within the school environment	Interviews with principals, teachers, and LSAs
		Equip the school health team with the skills and knowledge required to use the screening tool	(a) Train school health team on CAMH identification and use of screening tool (b) Distribute screening tools and guidelines	(a) Equipped school health team (b) Functional tools and guidelines	(a) Number of trained school health nurses (b) Number of tools and guidelines distributed	(a) Completed training registers (b) Signed sheets for tools and guidelines received	School health team are equipped to identify CAMH conditions and screen within the school environment	Improved CAMH screening within the school environment	Interviews with the school health team
		Improve CAMH knowledge and screening practice among PHC workers. Equip (nurses, lay counselors, WBOTs) with the skills required to use the screening tool	(a) Train PHC workers on CAMH identification and use of screening tool (b) Develop and distribute screening tools and guidelines	(a) Equipped PHC workers (b) Functional tools and guidelines	(a) Number of trained PHC workers (b) Number of tools and guidelines distributed	(a) Completed training registers (b) Signed sheets for tools and guidelines received	PHC workers are equipped to identify CAMH conditions and screen at the PHC clinics and within the community	Improved CAMH screening and management at the community and PHC level	Interviews with PHC operational managers
1b	Routine community awareness and CAMH promotion	Intensify CAMH awareness and mental health promotion to improve CAMH literacy within the community	(a) Train community CAMH volunteers and NPOs to conduct routine awareness/campaign programs within the community (b) Develop awareness materials (c) Map out a strategic plan for a routine awareness program (d) Employ the use of social media platforms to disseminate CAMH information (e) Partner with media houses such as radio stations to disseminate CAMH information	(a) Equipped CAMH volunteers (b) Access to campaign materials (c) Functional strategic plan for a routine awareness program (d) Active use of social media platforms to disseminate CAMH information (e) partnership with media houses such as radio stations to disseminate CAMH information	(a) Number of days dedicated to CAMH door-door community awareness (b) Number of campaigns materials distributed (c) Number of strategic plan documents distributed (d) Number of social media platforms developed to disseminate CAMH information (e) Number of sponsored programs through the partnership	(a) Completed activity log (b) Signed sheets for campaign materials received (c) Signed sheets for strategic plan received (d)Visibility of social media platforms (e) Number of aired CAMH programs	(a) CAMH volunteers are equipped to conduct routine awareness/campaign programs within the community (b) Awareness materials are adequately disseminated (c) Compliance with the strategic plan for a routine awareness program (d) Consistent update on the CAMH social media platforms (e) Consistent airing of CAMH programs	(a) Efficient CAMH awareness/campaign programs within the community (b) Improved CAMH awareness materials (c) Revised strategic plan for routine (d) Rebranded CAMH social media platforms (e) Increased number or airing time for CAMH programs	District mental health coordinator
2. Appropriate referral	Adequately define referral pathways	Design a functional district CAMH referral system (consolidate the referral policies from all sectors DSD, DOH, DBE)	(a) Design a referral guideline involving all the sectors and other possible identification sites	A well-defined referral system	Number of distributed referral guidelines	Schools’, PHC and hospital referral registers	CAMH conditions are appropriately referred to the site of care	Improved referral system	DMHC
		Adequately communicate and educate all stakeholders about the referral system	Train stakeholders from all sectors on the appropriate referral pathways	Stakeholders are adequately informed about the appropriate referral pathways	Number and categories of stakeholders trained	Completed training registers	Stakeholders are adequately equipped to refer CAMH cases appropriately	Improved referral system	DMHC
3.CAMH care package	(a) Design a CAMH care package	(1) Empower all care providers through training (2) Empower parents & families through education and support visits (3) Clearly define the CAMH care package for each condition (4) Improve treatment initiation process at the PHC level	Educate all care providers about the CAMH care packages	A well-designed CAMH care package	Number of care providers trained	Completed training registers	Stakeholders are adequately informed about the CAMH care packages	A well-designed CAMH care package	DMHC
	(b) Socio-economic support	(1)Create a support group for caregivers (2)Create a support group for adolescents living with CAMH conditions (3)Facilitate child support grant (4)Family Strengthening interventions	(1)Provide resources to facilitate the development of a support group for caregivers (2)provide resources to facilitate the development of a support group for adolescents living with CAMH conditions (3)develop a system to facilitate the disbursement of child support grant (4)Design family strengthening interventions	(1) A functional caregivers support group (2)A functional adolescent support group (3)A functional system of child support grant disbursement (4)Ongoing implementation of family strengthening interventions	(1) Number of support groups developed for caregivers (2)Number of support groups developed for adolescents (3)Number of child support grant disbursed (4)Number of functional interventions implemented	(1)Support group meeting attendance register (2)Support group meeting attendance register (3)Evidence of grant disbursed by DSD (4)Evaluation of the interventions	(1)Adequate and consistent support group meetings (2) Adequate and consistent support group meetings (3)Increased number of children on the disability grant (4)Adequately implemented interventions	(1)Strengthened support group (2) Strengthened support group (3)Improved system of grant disbursement (4)Improved interventions	Caregiver/ DSD/DoH
4a. Role clarification	Strengthen CAMH management system	(a) Clearly define the roles of different stakeholders from the different sectors (b) Design interventions to expand CAMH workforce through training, task-shifting/sharing, and supervision	(a) Organize intersectoral role clarification training for all stakeholders (b) Facilitate a task-sharing and supervision system between specialists and non-specialists (psychologists—Lay counselors, LSAs, school counselors, psychiatrist—medical officers and CAMH trained psychiatric nurses—psychiatric nurses, school health nurses)	(a) Stakeholders’ roles are adequately defined (b) A well developed and functional system of task-sharing and supervision	(a) Number and categories of stakeholders trained (b) Number of specialists and non-specialists enrolled in the system	(a) Completed training registers (b) Number of documented task-sharing/supervision activities	a) Stakeholders are adequately informed about their roles b) Adequate implementation of a task-sharing and supervision system	a) Clearly defined roles b) Improved supervision and task sharing system	DMHC
4b. Intersectoral governance	Intersectoral collaboration to achieve joint budgeting, design strategic plans, and collaborative services and stewardship	A coordinated system of collaboration	Creation of a CAMH team with representatives from DoH, DSD, DBE and other sectors	A functional intersectoral CAMH board	Number of departments represented in the board	Board meeting register and activity log	Increased number of CAMH intersectoral joint activities	Strengthened CAMH intersectoral activities	DOH, DSD, DBE

### Interventions/strategies for early identification of CAMH conditions

At the community level, proposed interventions for early identification include (i) the development of screening tools and improvement of screening quality, with service providers needing to be trained to ensure the effective use of the screening tools and to improve the quality of screening; (ii) educating the community on CAMH disorders and their symptoms through community awareness activities facilitated by NPOs. At the primary health care level, proposed interventions for early identification included efforts to (i) improve developmental screening by advocating for increased attention to developmental milestones and teaching mothers how to interpret "red flags" highlighted in the road to health cards; (ii) improving the quality of CAMH assessment by strengthening CAMH consultations with the available psychiatric nurses in the district. At the hospital level, interventions would include efforts to facilitate (v) timeous CAMH assessment and diagnosis through advocacy for prioritisation of CAMH cases to achieve prompt specialist interventions.

### Appropriate referrals and prompt access to CAMH services

At the community level, interventions to improve referral would include efforts to educate the community on CAMH health-seeking and appropriate treatment sites, with PHC facilities highlighted as the first point of call for CAMH care, from where onward referral to CAMH nurses, educational psychologists, and occupational therapists could occur. The development of a psychoeducation tool was identified as being important to assist community health workers in educating the community. At the PHC level, interventions identified includes placement of trained CAMH nurses in larger PHC facilities—such as Community Health Centres, with peer consultation and specialist supervision being solicited to further strengthen CAMH nurses' consultations. At the hospital level, interventions identified included clear referral pathways to and between specialists (clinical psychologists, psychiatrists, occupational therapists, and speech therapists), including referral and back referral procedures within the hospital system. Identified cross-cutting interventions included (i) the design of a clear multisectoral CAMH referral system; and (ii) adequate communication of referral pathways within sectors.

### Development of CAMH promotion, care and treatment packages

At the community level, identified efforts to develop effective CAMH care packages included designing community-based interventions such as parenting programs, family strengthening programs, awareness campaigns, school awareness and dropout prevention programs. At the primary healthcare level, interventions identified included (i) initiating psychosocial support for children/adolescents and caregivers by incorporating psychosocial support and youth-friendly services into primary health care; (ii) encouraging treatment initiation at the PHC level by capacitating clinic staff/psychiatric nurses in CAMH conditions through training and supervision; and (iii) the development of training packages for care providers to educate families and caregivers on CAMH conditions, as well as providing follow up and support services and develop treatment plans for different CAMH conditions.

### Intersectoral governance

The absence of systems aimed at tackling major CAMH issues and aligning all the available policies and programmes across all the government and non-government sectors was identified as the causal factors for the lack of intersectoral collaboration. Therefore, it is imperative to develop interventions and strategies that can help establish and build relationships across all the sectors. The identified strategies include (i) initiating intersectoral CAMH provincial and district policy development; (ii) initiating intersectoral CAMH data system development; (iii) establishing a district multisectoral CAMH working group; (iv) clearly defining the roles of care providers and specialists across all sectors, and (v) encouraging joint budgeting that adequately costs CAMH packages according to each sectors' contributions.

### Results from stage three

The stakeholders confirmed all the incorporated strategies as context-appropriate and highlighted strategies that could facilitate prompt identifications within the community and school environment as the first step to strengthening CAMH services in the district. The four major strategies included (i) developing CAMH community awareness programmes and psychoeducation interventions; (ii) developing a screening tool that non-specialist healthcare providers and educators can use to identify common CAMH disorders within the community; (iii) providing CAMH training for educators across the elementary, primary and secondary school phases. The training should focus on psychoeducation around CAMH disorders, appropriate use of the screening tools and the relevant referral pathways for the different conditions; (iv) defining clear pathways to care for different CAMH conditions and adequately communicating the pathways across all the sectors providing CAMH care.

The participatory workshops allowed the stakeholders to critically engage with CAMH services challenges and develop possible solutions. They acknowledged that implementing these strategies requires particular processes, including obtaining stakeholders buy-in across all the sectors, identifying strategic capabilities (existing resources within each departments) and adequately clarifying each departments’ roles to maximise resources and identifying priority intervention settings. Also, the stakeholders identified the need to promote collaboration and across all the sectors to strengthen CAMH services in the district. The stakeholders agreed to develop a dedicated forum for CAMH deliberations mainly to steer the implementation of the strategies and facilitate the development of local constituencies for change within each department. 

### Next steps

The purpose of conducting the participatory workshops was to identify and discuss the strategies, which is the first step towards the development of a district CAMH plan. There was no consideration of practical implications regarding relevant resources (including human resources), cost implications and relative cost-effectiveness. However, the strategies were developed to strengthen and maximise the available resources within the community, schools and the various departments to minimise cost and ensure cost-effectiveness. The next phase of the study will focus on (i) developing intervention packages, (ii) creating new positions to implement the strategies, (iii) estimating the cost of each package and conducting cost–benefit analysis, (iv) obtaining the inputs of all the stakeholders on the costs and (v) developing a district plan and economic modelling. 

## Discussion

Considering the lack of provincial and district CAMH policy documents and implementation plans to complement the national 2003 CAMH policy in South Africa [33], this study sought to address this problem through engaging in a three-step process in one district (Amajuba) as a case study on how to assist in the development of district CAMH plans. Informed by bottlenecks in CAMH services identified by formative studies in Amajuba [5, 7], we sought to initially facilitate the identification of strategies with key stakeholders to address these bottlenecks. The information generated by this process, as well as the formative studies, were then used to develop a Theory of Change for Amajuba of how to improve CAMH outcomes, with the view to helping the district to develop a CAMH plan.

To address the problem of poor intersectoral collaboration in CAMH services, the development of an intersectoral working group was suggested. Intersectoral collaboration is essential to clarify roles and responsibilities of different sectors in the provision of CAMH services. It is also crucial to provide multisectoral CAMH care packages and achieve joint budgeting- which is key to improving CAMH service delivery and mental health outcomes in children and adolescents [32, 39].

The design of user-friendly CAMH screening tools to facilitate early identification in the community was identified as a key strategy required to address challenges relating to poor identification and assessment. Failure to promptly detect CAMH cases in the district was directly linked to low levels of CAMH literacy and the lack of user-friendly screening tools at the community level, particularly in schools. This corroborates the findings of previous studies which establish links between early identification and the availability of valid, brief and easy to use CAMH screening tools [22, 28, 35, 44]. The development of easy-to-administer CAMH screening tools, training and supporting the Ward-based Primary Health Care Outreach Teams (WBPHCOTs), the integrated school health program (ISHP) nurses, learner support agents (L.S.A.s) and educators in the use of these tools should promote early identification and referral of children and adolescents in need of mental health services [15].

To address the inappropriate referrals and treatment delays, the participants suggested that all referral policies in each department be adequately communicated and integrated to develop a functional district multisectoral CAMH referral system that will promote appropriate referral and prompt access CAMH services. Also, the need to adequately educate all stakeholders about the consolidated referral system was emphasised. These findings are similar to that of Porras-Javier et al. [39] and a systematic review of pathways to mental health services for young people conducted by MacDonald et al. [31].

In light of the severe shortage of CAMH specialists, particularly the shortage of educational psychologists and clinical psychologists, the participants suggested the adoption of a task-sharing approach with specialist supervision and mentoring of non-specialists to equip them with the necessary skills. Several studies have highlighted the significance of task-sharing in narrowing mental health treatment gaps and facilitating access (Hanlon et al. [47]; Hoeft et al. [48]; Padmanathan & De Silva, [49] particularly for delivering CAMH services in low resource settings. A study conducted by Desta et al. showed that empowering teachers with the required CAMH skills could facilitate early identification, appropriate referral, and prompt treatment of CAMH conditions Desta et al. [50].

The possibility of addressing the scarcity of CAMH interventions at the PHC level by integrating CAMH services into primary health care was emphasised. Previous studies have identified PHC as a core site for CAMH identification and treatment [2, 3, 6]. The delivery of prevention and promotion programs were also suggested as being an important aspect of PHC, with chill rooms at the community clinics suggested as feasible sites for delivering mental health interventions such as mentorship programs to young people in the community.

It is also crucial to tackle inadequate CAMH awareness in the communities resulting in low CAMH knowledge in the district. Participants suggested that initiatives steered at increasing routine awareness campaigns in the community to sensitise parents, teachers, youths, and health professionals could improve CAMH knowledge and service outcomes in the district. A study conducted in nine countries by Hoven et al. [23] revealed improved CAMH knowledge and an increased willingness to engage in CAMH discussions among parents, teachers, and children after conducting awareness campaigns.

## Limitations of the study

We acknowledge that the study was only conducted in one district—therefore, the findings are not generalisable. However, the study provides an insight into how participatory methods can be used to identify and address CAMH service bottlenecks and how local stakeholders can collaborate to develop a systematic and holistic approach to strengthen CAMH services. This participatory approach could be used in developing strategies to enhance CAMH services in other contexts within the country and LMICs. The findings and recommendations described here are very much a work-in-progress and should be read as flexible guidelines that will be regularly updated in consultation with district stakeholders.

As with any approach, ToC has its limitations. The process of developing a ToC, particularly identifying detailed preconditions required to achieve the desired impact and deciding on suitable indicators to ascertain that the preconditions will produce the desired outcomes, is complex, expensive, and time-consuming [13, 16]. Hence, for this study, the researchers developed the actual ToC model using the information obtained from the formative studies and the strategies identified by the participants in the workshop. This approach is different from the standard approach to developing a ToC, where participants are facilitated to predict all the elements of the ToC in a workshop, which can be challenging. As De Silva et al. [17] highlighted, orientating stakeholders to identify, prioritise, measure key interventions, and predict the effect of particular strategies on the medium-term outcomes could be challenging due to time constraints. Our approach addressed these challenges and provided an opportunity for the researchers to critically reflect on the required ToC elements. The strategies identified in the workshop were then incorporated in the model–providing an alternative to the standard ToC development approach that can be adopted in busy health care settings.

Our study is still in the early stages of a sequential process. We have co-developed a ToC map in collaboration with the stakeholders involved in delivering CAMH services in the case study district. The next phase will be to create an intersectoral working team to develop a district CAMH plan.

## Conclusions

The findings of this study present a process of developing a ToC in low resource settings and provides insight into areas in need of immediate action to facilitate the development of a district CAMH plan. The identified causal factors and possible solutions to the bottlenecks facilitated the development of a ToC, which could ease the development of a district CAMH plan and ensure the effectiveness and sustainability of CAMH services in the district. The next step will be creating a multisectoral district task team that will engage with the findings and drive the development and implementation of a comprehensive plan to strengthen CAMH services in the district. Many of the lessons learned from this study could be applicable in other districts in South Africa and Southern African settings.

## Data Availability

Data and material will be made available on request.

## Abbreviations

CAMH: Child and Adolescent Mental Health
CMED: Community Mental Health Education And Detection
DBE: Department Of Basic Education
DOH: Department Of Health
DSD: Department Of Social Development
ISHP: The Integrated School Health Program
LMICs: Low- And Middle-Income Countries
L.S.A.s: Learner Support Agents
MhINT: Mental Health INTegration
NPOs: Non-Profit Organisations
psy-OPD: Psychiatric Outpatient Department
SANCA: South African National Council On Alcoholism
SIAS: Screening, Identification, Assessment, And Support
ToC: Theory Of Change
WBPHCOTs: Ward-Based Primary Health Care Outreach Teams
